# Evaluation of eating and rumination behaviour in cows using a noseband pressure sensor

**DOI:** 10.1186/1746-6148-9-164

**Published:** 2013-08-13

**Authors:** Ueli Braun, Luzia Trösch, Franz Nydegger, Michael Hässig

**Affiliations:** 1 Department of Farm Animals, Vetsuisse-Faculty, University of Zurich, Winterthurerstrasse 260, CH-8057 Zurich, Switzerland; 2 Agroscope Reckenholz-Tänikon ART, CH-8356 Ettenhausen, Switzerland

**Keywords:** Cattle, Eating and rumination behaviour, Automated recording

## Abstract

**Background:**

An automated technique for recording eating and rumination behaviour was evaluated in ten lactating Brown Swiss cows by comparing data obtained from a pressure sensor with data obtained via direct observation over a 24-hour period. The recording device involved a pressure sensor integrated in the noseband of a halter. The analysed variables included number and duration of individual rumination, eating and resting phases, total daily length of these phases and number of cuds chewed per day.

**Results:**

Eating and rumination phases were readily differentiated based on characteristic pressure profiles. Chewing movements during rumination were regular and generated regular waveforms with uniform amplitudes, whereas eating generated irregular waveforms with variable amplitudes. There was complete or almost complete agreement and no significant differences between data obtained via direct observation and pressure sensor technique. Both methods yielded an average of 16 daily eating phases with a mean duration of 28.3 minutes. Total time spent eating was 445.0 minutes for direct observation and 445.4 minutes for the pressure sensor technique. Both techniques recorded an average of 13.3 rumination phases with a mean duration of 30.3 (direct observation) and of 30.2 (pressure sensor) minutes. Total time spent ruminating per day, number of cuds per day and chewing cycles per cud were 389.3 and 388.3 minutes, 410.1 and 410.0 and 60.0 and 60.3 for direct observation and pressure sensor technique, respectively. There was a significant difference between the two methods with respect to mean number of chewing cycles per day (24′669, direct observation vs. 24′751, pressure sensor, P < 0.05, paired *t*-test). There were strong correlations between the two recording methods with correlation coefficients ranging from 0.98 to 1.00.

**Conclusions:**

The results confirmed that measurements of eating and rumination variables obtained via the pressure sensor technique are in excellent agreement with data obtained via direct observation.

## Background

Eating and rumination are quintessential activities of dairy cows, and observing these behaviours provides useful information regarding the cows’ health. A certain level of well being is a prerequisite for rumination [1]; excitement and stress [2], states of anxiety [3] and various diseases [4,5] inhibit rumination. Eating and rumination behaviour of sick cattle are of primary importance from a clinician’s standpoint. These activities are routinely monitored in sick cows during and after treatment. The time required for normalisation of eating and rumination in a sick animal has prognostic value and may be a reflection of the effectiveness of treatment. Observing eating and rumination behaviour of individual animals is difficult in large herds, especially if specific information regarding the duration of these behaviours, number of eructated cuds per unit of time or the number of chewing cycles per cud is sought. A novel device was recently developed for the detailed assessment of eating and rumination behaviour of cows [6-8]. It consists of a halter with a data logger incorporated in the noseband for the recording of jaw movements through a pressure sensor. Validation of this measuring technique was limited to the direct observation of two cows for three hours [6]. The goal of the present study was to evaluate data obtained from this device by comparing eating and rumination phases recorded by the data logger and by direct observation during a 24-hour period in ten cows.

## Methods

### Animals

Ten clinically healthy Brown Swiss cows aged 2.5 to 6.1 years (mean ± sd = 4.2 ± 1.3 years) and producing 20 to 25 kg (22.3 ± 2.0 kg) of milk per day were used for the study. The cows were 79 to 305 days (168.4 ± 72.5 days) post partum and were open or a maximum of 213 days (99.6 ± 73.3 days) pregnant. They weighed 580 to 730 kg (664 ± 38 kg).

### Housing and feeding

The cows were housed in tie-stalls, bedded with straw and had free access to water. They were milked twice daily. Hay was fed ad libitum starting 48 hours before the start of the study and continued until the end of the study. They also received 4.2 kg concentrate consisting of 2.2 kg corn pellets (Landi, Schneisingen) and 1.5 kg and 0.5 kg of a 17% and 39% protein mix, respectively (UFA, Lenzburg, Switzerland) twice daily. Orts were removed daily and the manger was cleaned.

### Clinical examination

All the cows underwent a physical examination, which included determination of general condition and demeanour, rectal temperature, heart and respiratory rates and rumen fill, layering and motility. Tests for reticular foreign bodies, swinging and percussion auscultation of the abdomen, rectal examination, urinalysis (colour, transparency, urine test strip and specific gravity) and evaluation of rumen juice, obtained via a stomach tube, (colour, odour, consistency, pH, methylene blue reduction test and chloride concentration) were also carried out. The results of the clinical examinations were described in detail [9].

### Pressure transducer

**Figure 1:**
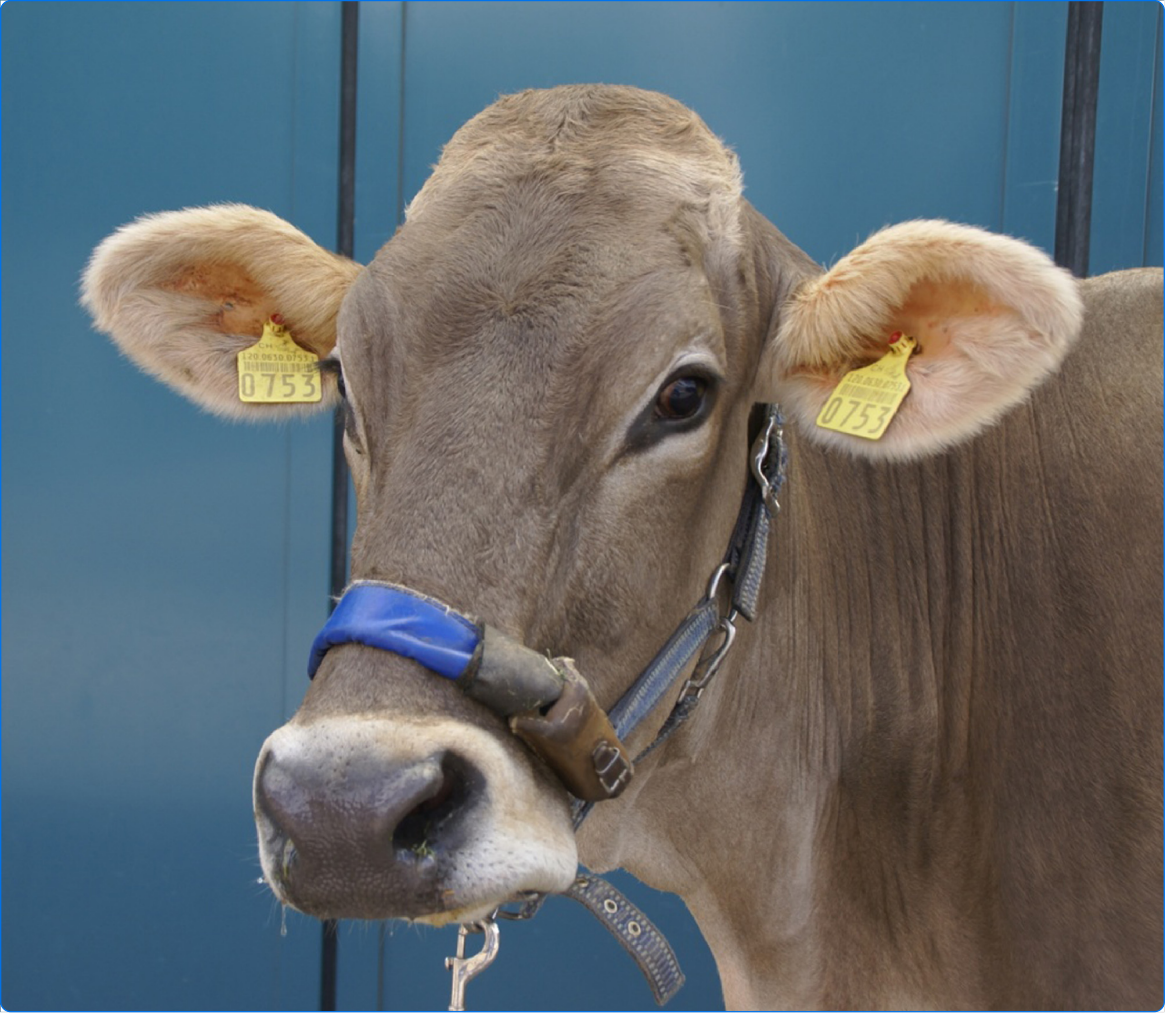
**Cow with recording halter.** Cow fitted with a recording halter incorporating an oil-filled tube and pressure sensor. The brown leather pouch near the cheek band contains the data logger.

**Figure 2:**
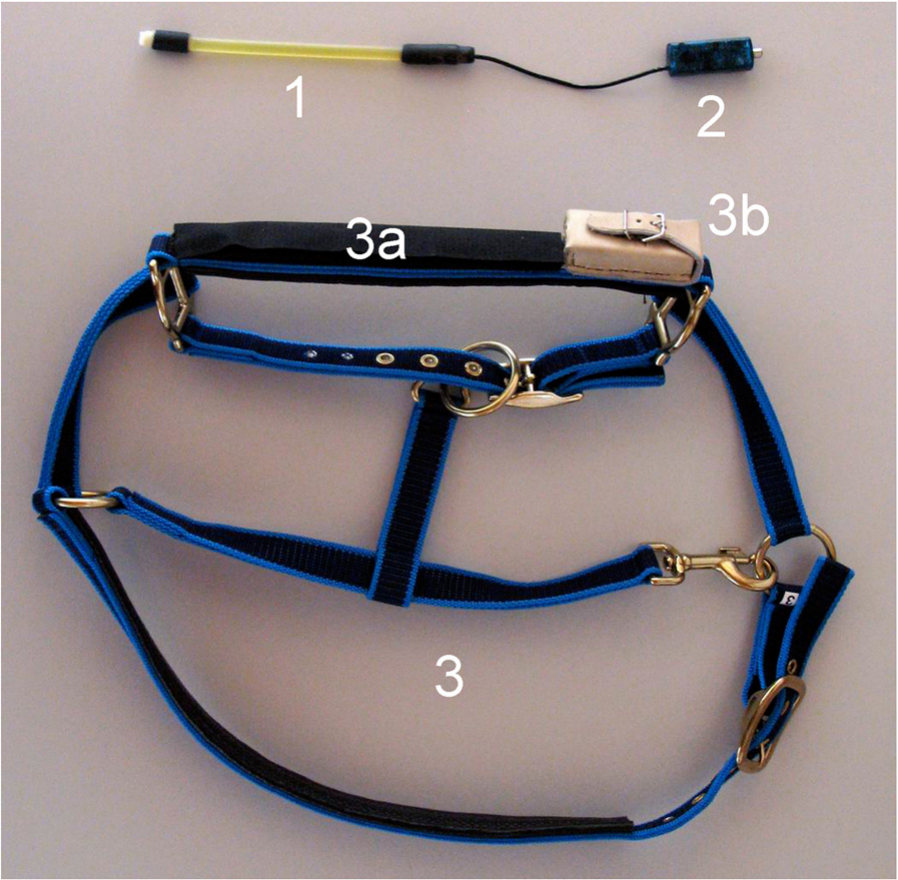
**Recording halter.** Recording halter for the investigation of eating and rumination behaviour in cows. **1** Oil-filled tube contains the pressure sensor and is integrated in the noseband, **2** USB logger, **3** Halter with noseband **(3a)** and leather pouch for data logger **(3b)**.

The device used in the study has been recently described (Nydegger et al. 2011a,b) and involved a pressure-sensitive sensor mounted on the noseband of a halter (MSR Electronics, Seuzach) (Figure 1). The method was developed and patented by ART and MSR Electronics (Patent CH 700 494 B1). The sensor, which picks up jaw movements, is mounted on the noseband of the halter and registers pressure changes in an oil-filled tube. Opening of the mouth causes bending of the tube and increases pressure within it. The change in mechanical pressure alters the electrical resistance in the sensor, which is recorded as a signal. Data were stored in a data logger (MSR 145 W, MSR Electronics), which was secured to the side of the halter in a leather pouch (Figure 2). The logger with a storage capacity of two million measurements was connected to the external pressure transducer and recorded the physical measurements. At the end of the examination period, the data were uploaded from the data logger to a personal computer (PC) via a USB cable. MSR-PC software (MSR Electronics) was used for data analysis.

### Pressure transducer recordings and direct observation

The cows were fitted with the recording halter at 8:00 hours on the day of examination. Periods of eating and rumination were then recorded via the pressure sensor as well as by simultaneous direct observation for 24 hours. Direct observation was done by three people, who alternated every four hours. The observer sat three metres in front of the cow and constantly watched one cow per observation session. Activities such as rumination, eating, drinking, grooming, vocalization, scratching, hierarchal behaviour and movements to keep flies away were recorded every 60 seconds. The number of chewing cycles per cud were determined with a mechanical hand counter. The recording halter was removed after 24 hours and the results were uploaded to a PC from the data logger for analysis. The periods of eating and rumination recorded by the pressure transducer were compared with those recorded by direct observation.

For each cow, the following variables were determined from the uploaded data and from direct observation by manual counting or by derivation from the time axis:

–  Number of rumination, eating and resting phases

– Duration of individual rumination, eating and resting phases

– Total length of rumination, eating and resting phases

– Number of regurgitated cuds per day

– Number of chewing cycles per cud and per day.

### Statistical analysis

The STATA 12 software (StataCorp LP, College Station, Texas, USA, 2011) was used for calculation of the mean, standard deviation and median, and the Wilk Shapiro test was used to test for normality. Results of direct observation and logger data were compared using a paired t-test. A value of P ≤ 0.05 was considered significant.

### Approval of the study by an ethical committe

The study was approved by an ethical committee of the canton of Zurich, Switzerland (Number 2010/41).

## Results

### Pressure patterns during eating and rumination

**Figure 3:**
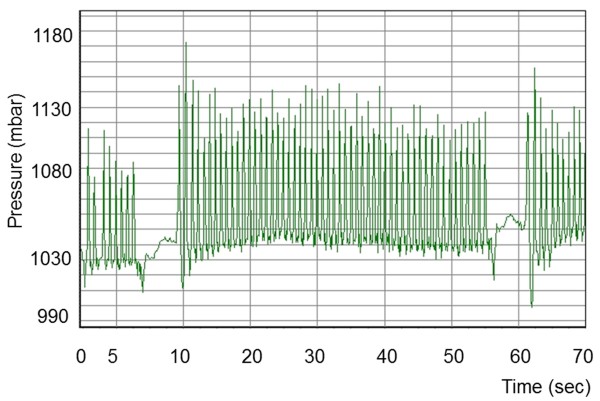
**Pressure profile during rumination.** Pressure profile recorded in a seven-year-old cow during rumination. The short intervals without pressure fluctuations (no jaw movements) and the uniformity of the waveform are characteristic of rumination. Seconds after start of measurement.

**Figure 4:**
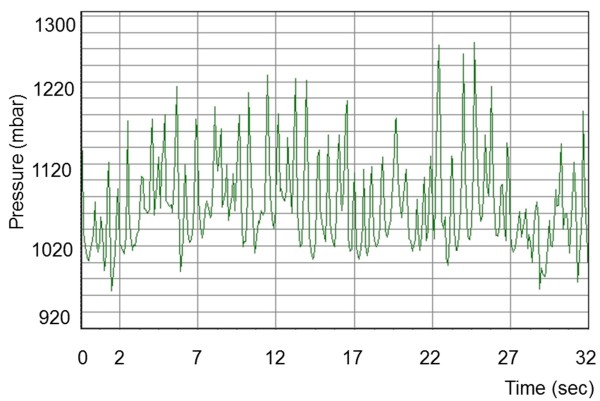
**Pressure profile during eating.** Pressure profile recorded in a four-year-old cow during eating. There is considerable variation of the pressure amplitudes resulting in an irregular waveform.

Eating and rumination could be readily differentiated based on characteristic pressure profiles. Each chewing cycle was associated with a peak on the pressure profile. Rumination consisted of a series of uniform chewing movements (Figure 3) that created a regular waveform. This regular profile pattern was briefly interrupted by periods without jaw movements when the cow swallowed the cud and regurgitated a new one. The pressure pattern recorded during eating was much more irregular and created an irregular waveform (Figure 4). The intervals between regurgitation of the cud also varied, and at times there were short intervals during which no chewing movements were recorded because of other activities such as pushing feed around in the manger. However, these intervals did not occur as regularly as those between rumination phases, when a cud is regurgitated.

### Eating

There were no significant differences in the results of direct observation and pressure sensor recordings during eating (Table 1). Both methods yielded a mean of 16 eating phases per day. Each phase lasted a mean of 28.3 minutes, and the total time spent eating per day was 445.0 minutes for direct observation and 445.4 minutes for the pressure sensor technique.

**Table 1:** Number and duration of individual eating phases and total time spent eating per day

**Variable**	**M**	**n**	**Mean**	**sd**	**Min.**	**Max.**
Number of eating phases	D	10	16	1.9	12	19
	P	10	16	1.9	12	19
Duration of eating phases (min)	D	10	28.3	5.3	22.5	40.0
	P	10	28.3	5.5	22.0	40.7
Total time spent eating (min)	D	10	445.0	44.7	373	505
	P	10	445.4	44.5	375	497

M Measuring technique, D Direct observation, P Pressure sensor technique.

### Rumination

There was no significant difference in the results of direct observation and pressure sensor recordings with regard to the number, duration and total length of rumination phases (Table 2). Both methods yielded 13.3 rumination phases, which had mean durations of 30.2 minutes (direct observation) and 30.3 minutes (pressure transducer). The mean total length of rumination was 389.3 minutes for direct observation and 388.3 minutes for the pressure sensor technique. The number of regurgitated cuds was 410.1 per day for direct observation and 410.0 for the pressure sensor technique, and the number of chewing cycles per cud was 60.0 for direct observation and 60.3 for the pressure sensor technique (Table 3). The mean number of daily chewing cycles differed significantly between the two recording methods; there was a mean of 24′669 cycles for direct observation and 24′751 cycles for the pressure sensor (P < 0.05, paired *t*-test).

**Table 2:** Number and duration of individual rumination phases and total time spent ruminating per day

**Variable**	**M**	**n**	**Mean**	**sd**	**Min.**	**Max.**
Number of rumination phases	D	10	13.3	2.8	10	20
	P	10	13.3	2.8	10	20
Duration of ruminating phases (min)	D	10	30.3	2.2	18.5	38.6
	P	10	30.2	2.1	18.6	38.0
Total time spent ruminating (min)	D	10	389.3	50.6	284	454
	P	10	388.3	50.9	278	447

M Measuring technique, D Direct observation, P Pressure sensor technique.

**Table 3:** Number of cuds per day and number of chewing cycles per cud and per day during rumination

**Variable**	**M**	**n**	**Mean**	**sd**	**Min.**	**Max.**
Number of cuds per day	D	10	410.1	46.9	348	478
	P	10	410.0	47.1	347	478
Number of chewing cycles per cud	D	10	60.0	2.7	43.3	69.1
	P	10	60.3	2.6	44.3	69.4
Number of chewing cycles per day during rumination	D	10	24*'*669 ^*^	4*'*833	15*'*057	31*'*770
	P	10	24*'*751	4*'*789	15*'*369	31*'*893

M Measuring technique, D Direct observation, P Pressure sensor technique.

^*^Difference between measuring techniques, P < 0.05 (paired *t*-test).

### Resting and other activities

**Figure 5:**
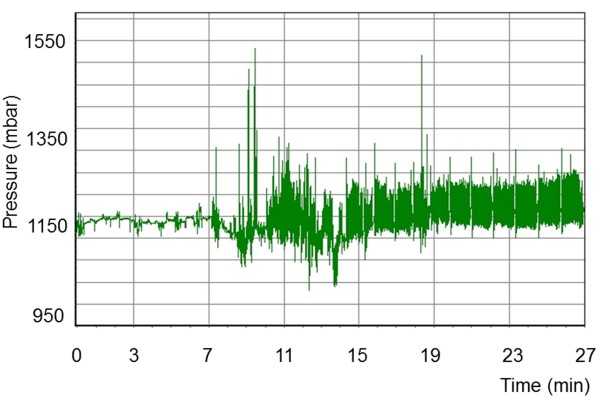
**Pressure profile during resting, eating and rumination.** Pressure profile recorded in a 5.5-year-old cow during resting, eating and rumination. No jaw movements were registered during the first seven minutes, after which the cow started eating and (after 15 minutes) ruminating.

**Figure 6:**
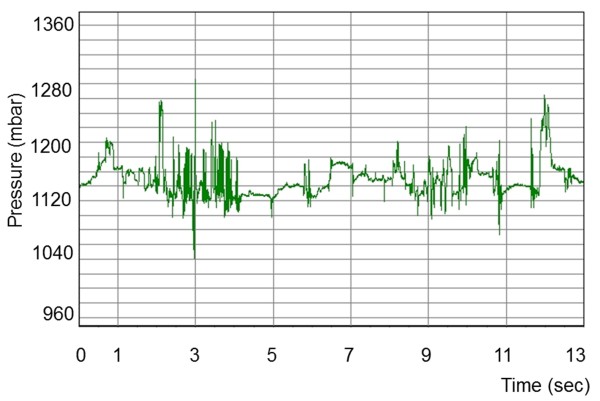
**Pressure profile during other activities.** Pressure profile recorded in a three-year-old cow during other activities. The pressure fluctuations during the first four minutes occurred during grooming behaviour and the fluctuations from 8 to 12 minutes during drinking.

Resting was characterised by extended periods of time without jaw movements (Figure 5). Pressure patterns other than those recorded during eating, rumination and resting were attributed to other activities, which did not generate regular pressure patterns (Figure 6). A variety of pressures ranging from low to high were recorded during activities such as scratching, hierarchal behaviours or drinking. There was no significant difference between the two recording methods with respect to the number of resting phases, duration of individual resting phases and total length of resting phases (Table 4). The results were very similar; both methods yielded a mean of 26.1 resting phases of 23.5 minutes duration in 24 hours. The total length of resting was a mean of 605.7 minutes for direct observation and 604.2 minutes for the pressure sensor technique.

**Table 4:** Number and duration of resting phases

**Variable**	**M**	**n**	**Mean**	**sd**	**Min.**	**Max.**
Number of resting phases	D	10	26.1	3.3	22	32
	P	10	26.1	3.3	22	32
Duration of resting phases (min)	D	10	23.5	1.9	18.2	30.6
	P	10	23.5	1.2	18.2	30.8
Total time spent resting (min)	D	10	605.7	63.9	506	693
	P	10	604.2	64.4	505	687

M Measuring technique, D Direct observation, P Pressure sensor technique.

### Correlation between data from direct observation and pressure sensor technique

There were very strong correlations between the two recording methods for eating, ruminating and resting times as well as the number and duration of eating, rumination and resting phases. The correlation coefficients were 0.98 (total length of eating), 0.99 (duration of eating, rumination and resting phases, total length of rumination and resting) and 1.00 (number of eating, rumination and resting phases).

## Discussion

The recording halter used in this study was easy to put on the cows, was well tolerated and proved to be reliable and robust. The padded and adjustable noseband and head piece were comfortable for the cows and guaranteed a perfect fit. The halter did not seem to affect the normal behaviour of the cows. The fit of the halter did not affect pressure patterns except for pressure amplitude. The most useful pressure profiles were obtained when the halter allowed placement of a hand between the bridge of the nose and noseband. Compared with devices that are based on acoustic sensors and are therefore only useful for recording rumination behaviour [10,11], the greatest advantage of this device was its versatility. It allowed simultaneous measurement of several variables including number and duration of individual rumination, eating and resting phases, total daily length of these phases, number of regurgitated cuds per day, number of chewing cycles per cud and the total daily number of chewing cycles during eating and rumination. Rumination phases were most easily recognised based on the regular chewing movements and the regular intervals at which they occurred. The unmistakable pressure pattern was even apparent when cows made scratching movements or attempts to keep flies away during rumination. Likewise, eating phases were easily identified even though the pressure pattern and waveform were less regular than during rumination. Both rumination and eating behaviour could be reliably differentiated from other activities using this device.

Comparison of data from the pressure sensor technique and direct observation revealed complete agreement with respect to the number of rumination, eating and resting phases. Small differences between the two techniques with respect to the durations of these phases and the total daily lengths of these behaviours were not statistically significant. The mean number of regurgitated cuds per day also did not differ between the two techniques, and in only one cow did direct observation yield one cud more than the pressure sensor technique. The only significant difference between the two methods was calculated for the mean number of daily chewing cycles, which was greater for the pressure sensor technique than for direct observation (24′751 versus 24′669). The largest difference recorded in any of the cows was 312 cycles. With the direct observation technique, the number of chewing cycles was determined by manually counting the peaks in the recordings. This is very reliable although small counting errors are possible, in agreement with a validation study of another device [12]. It is possible that jaw movements are missed when direct view of the jaw or muzzle is obscured by movement of the head. This small discrepancy between techniques notwithstanding, a difference of 312 chewing cycles amounts to about 1.25% and is considered clinically irrelevant. Our results allow the conclusion that the agreement between data obtained from the pressure sensor technique and direct observation is approximately 98.8%.

There was good overall agreement between our data and those published earlier. The number of eating phases varied from 12 to 19 with a daily mean of 16, compared with 12 [13] and up to 20 phases [14]. The total length of eating ranged from 6.3 to 8.3 hours (375 to 497 minutes) with a mean of 7.4 hours (445 minutes), which was longer than results reported in the 1960s of 4.0 [15] and 4.2 hours [16] in cows fed hay *ad libitum*. Other authors reported daily eating times of approximately 5.5 hours [13,17], 4 to 9 [18], 4 to 7 [14] and 4 to 12 hours [19]. A likely reason for the shorter eating times in the 1960s is the much smaller production level at that time and the smaller body size of cows. The modern dairy cow is bred for high milk yield, which necessitates an increased feed intake and thus longer eating times. With each additional kg of milk produced, mean dry matter intake increases by 0.16 kg [20], and with each 100 kg increase in live weight, mean dry matter intake increases by 0.34 to 2.00 kg [21-23]. Studies of the relationship between eating time and hay particle size have produced conflicting results. Cows fed alfalfa hay with a particle size of 30 mm had longer eating times than cows fed the same hay with a particle size of 15 mm [24], whereas time spent eating, time spent ruminating and total time spent chewing were not significantly different in cows fed hay with particles measuring 5.40, 8.96 and 77.90 mm [25]. On the other hand, eating minutes per kilogram dry matter intake and neutral detergent fibre intake tended to increase linearly as forage length increased [25]. The number of daily rumination phases of 10 to 20 observed in this study was in agreement with published numbers [14,18,19]. The mean duration of a rumination phase was 30.2 minutes and thus slightly shorter than previously published values of 40 to 50 minutes [18]. The total length of rumination recorded by the pressure sensor technique varied from 4.6 to 7.5 hours (278 to 447 minutes) and the mean was 6.5 hours (388.3 minutes), which was in general agreement with values of 4 to 9 hours per day reported by others [14,18,19,26]. Beauchemin [14] indicates 10 h as a physiological limit. Others have observed that following periods of high feeding times and intakes, cows spent more time ruminating [27].

The number of regurgitated cuds per day ranged from 347 to 478, compared with 360 to 790 cuds reported previously [18]. The mean number of chewing cycles per cud was 60.3 with a range of 44 to 69, compared with 52 [17] and from 40 to 60 cycles [18] reported by others. This number increases with increasing fibre content of the feed [14]. The number of chewing cycles per cow varied greatly from 15′369 to 31′893 but the mean of 24′751 was in good agreement with 26′400 cycles reported previously [18].

## Conclusions

The results of the present study in cows with a milk yield of 20 to 25 kg and fed hay ad libitum and 4.2 kg concentrate confirmed that measurements of eating and rumination variables obtained via the pressure sensor technique are in excellent agreement with data obtained via direct observation. This novel recording device is therefore well suited for in-depth study of eating and rumination behaviour in cows. The reference values established in this study should be corroborated by measurements in larger numbers of cows of different breeds, different production levels and different diets.

## Competing interests

The authors declare that they have no competing interests.

## Authors’ contributions

UB initiated, planned and supervised the study, and he wrote the manuscript, LT performed the study, FN planned the study and MH did the statistical evaluation. All authors have read and approved the manuscript.

## Sources of fundings

This study was financed by the University of Zurich, Switzerland.
